# Synergistic Efficiency of a Novel Temperate Phage YF1204 and Amikacin Against Carbapenem-Resistant *Pseudomonas aeruginosa* and Its Biofilms

**DOI:** 10.3390/microorganisms14030549

**Published:** 2026-02-27

**Authors:** Yinfeng Yang, Noura M. Bin Yahia, Yafei Pan, Zhaoxia Ran, Jing Yang, Yanhui Yang, Gang Li

**Affiliations:** 1 Center of Medical Laboratory, General Hospital of Ningxia Medical University, Ningxia Key Laboratory of Clinical and Pathogenic Microbiology, General Hospital of Ningxia Medical University, Yinchuan 750004, China; yangyinfeng0826@163.com (Y.Y.); pyf9267131996@163.com (Y.P.); rzx19921019@163.com (Z.R.); yangjing1420@163.com (J.Y.); 2 Ningxia Key Laboratory of Infectious and Immunity, School of Basic Medical Sciences, Ningxia Medical University, Ningxia Key Laboratory of Clinical and Pathogenic Microbiology, General Hospital of Ningxia Medical University, Yinchuan 750004, China; nourabinyahia@gmail.com

**Keywords:** *Pseudomonas aeruginosa*, temperate bacteriophage, phage-antibiotic synergy, amikacin, carbapenem resistance

## Abstract

Infections caused by carbapenem-resistant *Pseudomonas aeruginosa* (CRPA), especially chronic infections associated with biofilm formation, have become a major clinical challenge. Phage therapy has received much attention as an alternative strategy, but temperate phages have limited direct application due to their lysogenicity. The aim of this study was to explore the synergistic therapeutic effect of a novel temperate phage combined with antibiotics. A temperate *Pseudomonas* phage YF1204 was isolated from the patient’s bronchoalveolar lavage fluid and systematically characterized by whole-genome sequencing, transmission electron microscopy, and host range analysis. The synergistic antibacterial and anti-biofilm effects of phage with amikacin (AK) were evaluated by using the checkerboard test, a time-killing curve based on optical density (OD600) and crystal violet staining, and the cytocompatibility was analyzed by using the CCK-8 method. The results showed that phage YF1204 belonged to the *Siphoviridae* family and had typical temperate phage genome characteristics (containing integrase gene). It also showed lytic activity against 41.4% (87/210) of the clinical isolates, especially against carbapenem-resistant strains. When YF1204 was combined with AK, it reduced the minimum inhibitory concentration (MIC) of AK by 2- to 8-fold across all tested strains, respectively. Moreover, the inhibitory effect against CRPA was significantly enhanced (achieving suppression indexes about 80% ) and biofilm formation was inhibited with an inhibition ratio of 48.75%. Cell experiments showed that YF1204 had no significant toxicity to THP-1 cells. The combination of YF1204 and AK exhibited significant synergistic bactericidal and anti-biofilm activities, providing a novel therapeutic strategy with translational potential for CRPA-induced refractory infections.

## 1. Introduction

*Pseudomonas aeruginosa* (Pa) is a formidable opportunistic pathogen responsible for a wide spectrum of life-threatening healthcare-associated infections, including ventilator-associated pneumonia, bloodstream infections, and chronic wound and cystic fibrosis-related pulmonary diseases [1,2]. The emergence and dissemination of carbapenem-resistant *P. aeruginosa* (CRPA) have further narrowed the therapeutic window, often relegating clinicians to use last-resort antibiotics with limited efficacy and significant toxicity [3]. Compounding the challenge of planktonic bacteria, *P. aeruginosa* exhibits a profound capacity for forming biofilms, which confer up to a 1000-fold increase in antibiotic tolerance, and which are central to the pathogenesis of chronic, recalcitrant infections [4,5]. Consequently, there is an urgent, unmet clinical need for novel therapeutic strategies capable of eradicating drug-resistant *P. aeruginosa*.

Faced with this post-antibiotic era, the scientific community has witnessed a resurgence of interest in bacteriophage (phage) therapy as a promising alternative or adjunct to conventional antibiotics. Phages, the natural predators of bacteria, offer high specificity, the ability to self-replicate at infection sites, and enzymatic mechanisms to degrade biofilm matrices [6]. However, temperate phages are capable of entering a lysogenic cycle where their genome integrates into the host chromosome, rendering the bacterium a lysogen. This propensity for lysogeny raises significant concerns due to superinfection immunity, horizontal gene transfer, and the lack of immediate bactericidal activity and it has historically precluded their therapeutic use [7,8]. Despite the historical preference for strictly lytic phages in therapy, recent evidence has revealed that temperate phages can be therapeutically valuable when used in rationally designed combinations with antibiotics. In addition, temperate phages are more readily isolated from environmental and clinical sources than their strictly lytic counterparts, representing a vast, underexploited resource [9,10]. The key to harnessing their potential lies in strategies that circumvent or exploit the lysogenic lifecycle.

Recent strategies have sought to leverage the properties of temperate phages rather than avoid them. A promising approach involves combining temperate phages with sub-inhibitory concentrations of antibiotics, a phenomenon known as phage-antibiotic synergy (PAS) [11,12,13]. Notably, a novel form of specific to temperate phages (tPAS) has been described, wherein the fluoroquinolone antibiotic (e.g., ciprofloxacin) did not necessarily enhance phage replication but instead selectively targeted and eliminated lysogenized cells, preventing population regrowth and leading to profound bacterial eradication [11]. However, the mechanistic basis for synergy may differ substantially depending on the antibiotic class. Aminoglycosides such as amikacin target the bacterial ribosome, causing misreading of mRNA and production of misfolded, nonfunctional proteins [14,15]. This mechanism could interact with temperate phage biology through several pathways. Hence, the synergistic effect of temperate phage and amikacin on antibacterial performance deserves systematical investigation.

In this research, a novel temperate phage targeting *P. aeruginosa*, YF1204, was isolated from the bronchoalveolar lavage fluid of a tuberculous bronchiectasis patient in the respiratory critical care department of the General Hospital of Ningxia Medical University. Based on the above considerations, we hypothesized that the phage YF1204 might exhibit synergistic activity with antibiotics against CRPA, particularly with AK, given the observed susceptibility patterns in preliminary screening. Specifically, we aimed to: (i) characterize the genomic and biological properties of phage YF1204; (ii) determine its host range against a large collection of clinical *P. aeruginosa* isolates; (iii) test whether combinations of YF1204 with clinically relevant antibiotics, particularly AK, would yield enhanced antibacterial activity against both planktonic and biofilm-associated CRPA; and (iv) assess the preliminary safety profile of YF1204 on human cells. We anticipated that the combination of a temperate phage with an aminoglycoside antibiotic might exploit the vulnerabilities of the lysogenic state through mechanisms distinct from those described for DNA-damaging agents, potentially transforming a therapeutic drawback into a strategic advantage.

## 2. Materials and Methods

### 2.1. Bacterial Strains and Reagents

Clinical Pa strains, including CRPA, were isolated from the clinical laboratory at the General Hospital of Ningxia Medical University and preserved in 40% glycerol at −80 °C, which was approved by the Professional Ethics Committee of Clinical Research (KYLL-2025-2454). Antimicrobial susceptibility testing was performed using the VITEK 2 Compact system (bioMérieuxSA, Marcy-l’Étoile, France), and the results were interpreted according to the Clinical and Laboratory Standards Institute (CLSI) guidelines.

### 2.2. Isolation, Purification and Propagation of Phage YF1204

Phage YF1204 was isolated from clinical bronchoalveolar lavage fluid specimen using a modified enrichment protocol using a randomly selected clinical Pa strain as the host. Sputum and bronchoalveolar lavage fluid samples were incubated with a 4 mL phage buffer for 2 h at 37 °C on a shaking table and were centrifuged at 8000 rpm for 10 min. The supernatant was collected and filtered through a 0.22 μm filter to obtain phage stock solution. The phages were purified 6–8 times using the double-layer agarose plate method until a single type of plaque morphology was observed. Phage YF1204 was subsequently amplified and counted using the double-layer agarose plate method.

### 2.3. Morphology Analysis

Soft agarose gel blocks containing phage were incubated in phage buffer for 4 h at 25 °C, then centrifuged at 5000× *g* for 10 min to remove agarose and bacterial debris. The supernatant was filtered through a 0.22 μm filter to obtain crude phage suspension. Phage particles were then concentrated by ultracentrifugation at 35,000× *g* for 60 min at 4 °C. The pellet was resuspended in phage buffer, adsorbed onto 200 mesh carbon coated copper mesh, and stained with 2% phosphotungstic acid. Electron microscopic images were taken using a transmission electron microscope (TEM).

### 2.4. Phage Whole-Genome DNA Extraction, Sequencing and Analysis

The whole genome of phage YF1204 was extracted according to previous reference with a slight modification [16]. Whole-genome sequencing of phage YF1204 was performed with the use of an Illumina NovaSeq 6000 system (Illumina, San Diego, CA, USA). The host genome was removed using bowtie2 and samtools, and reassembled using SPAdes-3.15.3. The whole-genome sequence was uploaded to BLAST, and 2 similar phage sequences were selected and analyzed with Phage YF1204 via Easyfig (2.2.5) [17]. Based on the whole genome sequence similarity of the phage, the online website Viptree (genome.jp/viptree/upload, accessed on 12 June 2025) was used to compare the genomes of the phage and obtain the phylogenetic tree [18].

### 2.5. Optimal Multiplicity of Infection (MOI) and One-Step Growth Curve

A total of 100 μL of phage solution and 100 uL of host bacteria in logarithmic growth phase were added to the test tube to make the MOI (PFU/CFU) 0.001, 0.01, 0.1, 1 and 10, respectively. Subsequently, 5 mL of LB broth was added and incubated for 4 h at 37 °C with shaking at 200 rpm. Finally, the phage amplification solution was centrifuged (10,000 rpm, 10 min), and the supernatant was filtered through a 0.22 μm pore size Millipore filter (Merck Millipore Ltd., Carrigtwohill, Ireland) and diluted in a 10-fold gradient. The plaque forming unit (PFU) was determined by the double-layer agarose plate method. The MOI with the highest PFU was the optimal MOI of the phage YF1204 (*n* = 3).

One-step growth curves of phage YF1204 were evaluated with reference to previous methods, with slight modifications. In brief, phage YF1204 (MOI = 10) was incubated at 37 °C for 15 min to achieve adsorption. Subsequently, the samples were centrifuged at 12,000 rpm for 5 min at 4 °C and the supernatant was discarded. The precipitate was re-suspended in LB broth and incubated at 37 °C with shaking at 200 rpm for 120 min. Samples were collected every 10 min and the PFUs were quantified to generate growth curves. The burst size = (final PF − initial free PFU)/number of infected bacterial cells (*n* = 3).

### 2.6. Thermal and pH Stability

The thermal stability test was performed as follows: 100 μL phage suspension in 900 μL SM buffer was incubated for 1 h at different temperatures (4, 25, 37, 50, and 60 °C). The PFU of phage was determined by counting on a double layer agarose plate method (*n* = 3).

The pH stability test was carried out as follows: 100 μL of phage were suspended in 900 μL of SM buffer (pH 4.0–9.0), respectively, and kept for 1 h at 37 °C in a constant temperature incubator. Serial multiplier dilutions were then performed and PFU was immediately determined using the double-layer plate method (*n* = 3).

### 2.7. Host Range Assay

The host range of phage YF1204 was determined by a spot assay. Clinical Pa isolates in the logarithmic growth phase were adjusted to an OD_600_ of 0.5 using PBS, and bacterial suspensions were evenly distributed on LB agarose plates. Subsequently, 10 μL of phage YF1204 were immediately dropped to the surface of the bacterial layer and incubated overnight at 37 °C. The host range was assessed by observing plaque formation: complete for clear plaque, partial for light plaque, and negative for absence of plaque. All experiments were performed in triplicate to ensure consistency.

### 2.8. Evaluation of Antibacterial Effect

The antibacterial test was performed with slight modifications referring to the previous literature [19]. The frozen Pa cultures were incubated overnight at 37 °C with shaking at 220 rpm. Overnight cultures of bacterial strains were reverse diluted at a ratio of 1:200 and regrown for 3 h (to OD_600_ to 0.15 to 0.25). Then, the regrown cultures were diluted 1:100 (OD_600_ to 0.001–0.002) in 96-well plates. Depending on the experimental conditions, phages and antibiotics were added to achieve the target MOI and successive antibiotic concentrations (4×, 2×, MIC, 1/2, 1/4, 1/8, 1/16, 1/32, 1/64 MIC) to a total volume of 200 μL. The antibiotics selected were ceftazidime, meropenem, amikacin, ciprofloxacin and polymyxin B (Macklin, Shanghai, China). The final cultures on 96-well plates were then incubated at 37 °C for 18 h with shaking, OD_600_ was read every 30 min using a microplate reader (Synergy 2, BioTek, Winooski, VT, USA), and growth curves were plotted using GraphPad Prism 9.0. The suppression index (SI) was calculated as the area under the curve (AUC) of the control group minus the antibiotic-treated condition, phage-treated condition or phage-antibiotic condition, respectively, divided by the AUC of the control group.

### 2.9. Biofilm Formation Inhibition Assay

The Pa bacteria in the logarithmic growth phase were dispersed into MH liquid medium and prepared as a bacteria suspension with a concentration of approximately 1 × 10^8^ CFU/mL. The bacterial suspension was mixed with a specific concentration of phage alone (MOI = 10) or phage-1/8 MIC AK, and inoculated into a 6-well plate to a total volume of 2 mL. After gentle shaking, the suspension was placed into a 37 °C incubator for 24 h. After that, the culture medium was sucked out and gently washed at 37 °C in sterile PBS 3 times, and then fixed in 4% paraformaldehyde for 15 min. The paraformaldehyde was sucked out and gently washed in PBS 3 times before being dried naturally. Then, it was dyed with 0.1% crystal violet for 15 min. After staining, the residual staining solution was washed and dried naturally to observe the morphology of bacterial biofilms.

Crystal violet was dissolved in 75% ethanol for 5 min for decolorization quantitative analysis. A total of 150 μL of decolorization solution was transferred to a new 96-well plate, and the absorbance of the decolorization solution at a wavelength of 590 nm was recorded by a microplate reader (Synergy 2, BioTek, USA). Finally, the biofilm inhibition ratio was calculated by the formula (*n* = 3). The biofilm inhibition ratio (%) = (OD_control_ − OD_treated_)/OD_control_ × 100%.

### 2.10. Cytotoxicity Test of Phage YF1204

The cytocompatibility assessment was conducted in accordance with previous studies with slight modifications [20]. The THP-1 cell line was obtained from the Procell Biotechnology Co., Ltd. (Wuhan, China) and cultured in RPMI-1640 media (Procell, Wuhan) containing 10% fetalbovine serum (FBS, Procell, Wuhan, China), 1% penicillin and streptomycin (Solarbio, Beijing, China) and 0.05 mM β-mercaptoethanol (β-ME, Solarbio, Beijing). Firstly, the THP-1 cells were cultured at a density of 5 × 10^4^ cells per well in a 96-well plate and overnight at 37 °C with 5% CO_2_. Different concentrations of phage YF1204 (1000, 100, 10, 1 PFU/cell, respectively) were added and incubated with cells at 37 °C for 24 h. Following incubation, 10% Cell Counting Kit 8 (CCK-8) regent (Solarbio, Beijing) was added to each well. After an additional incubation for 2 h, absorbance at OD_450_ was detected using a multifunctional microplate reader (Synergy 2, BioTek, USA). The well without phage served as the control group, and each group of experiments was performed three times in parallel. The cell viability ratio was calculated according to the formula. The cell viability (%) = OD_phage_/OD_control_ × 100%.

### 2.11. Statistical Analysis

All data were presented as mean ± standard deviation (M ± SD) by at least triplicate values. All statistical analysis was performed using one-way ANOVA with GraphPad Prism 9.1 (USA); the *p* < 0.05 was marked statistically significant (* *p* < 0.05, ** *p* < 0.01, *** *p* < 0.001, **** *p* < 0.0001).

## 3. Results

### 3.1. Phage Morphology and Genome Analysis

#### 3.1.1. Isolation and Basic Characterization of Phage YF1204

**Figure 1:**
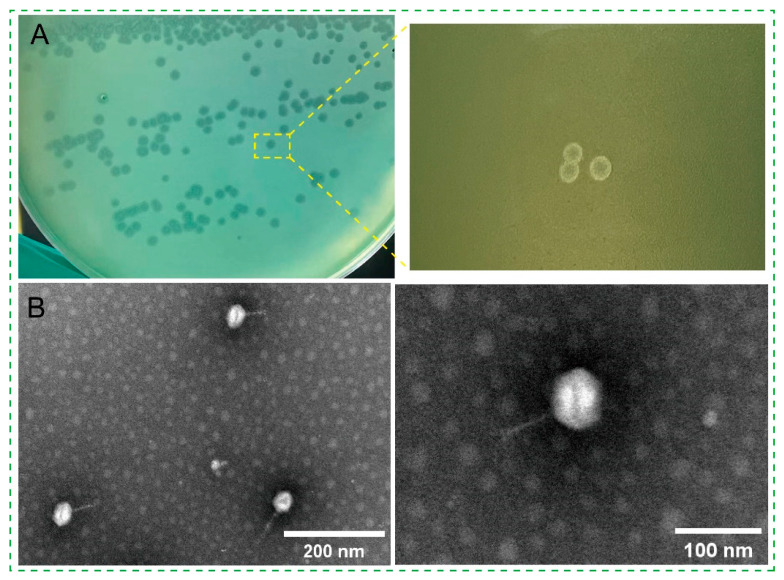
The morphology of phage YF1204. (**A**) Plaque morphology and (**B**) transmission electron microscopy morphology.

A temperate phage strain targeting *P. aeruginosa*, designated YF1204 (GenBank: PX587829), was isolated from the patient’s bronchoalveolar lavage fluid. Phage YF1204 formed turbid plaques with indistinct margins on lawns of the host (Figure 1A), a morphological trait commonly associated with temperate phages capable of lysogeny. The plaques measured approximately 1–2 mm in diameter after 18–24 h of incubation at 37 °C. The turbid center reflects the presence of lysogenized bacterial cells that are resistant to superinfection, consistent with a life cycle that includes both lytic and lysogenic pathways [21]. Transmission electron microscopy (TEM) revealed that the structural integrity and clearly visible head–tail connector support the classification of YF1204 as a tailed phage with typical *siphoviral* architecture characterized by icosahedral capsid of approximately 67 nm in diameter and a long, non-contractile tail about 69 nm in length (Figure 1B). This structural organization indicated that it belonged to the *Siphoviridae* family, which includes numerous temperate phages known to infect *Pseudomonas* species [13,22].

#### 3.1.2. Genomic Analysis of Phage YF1204

**Figure 2:**
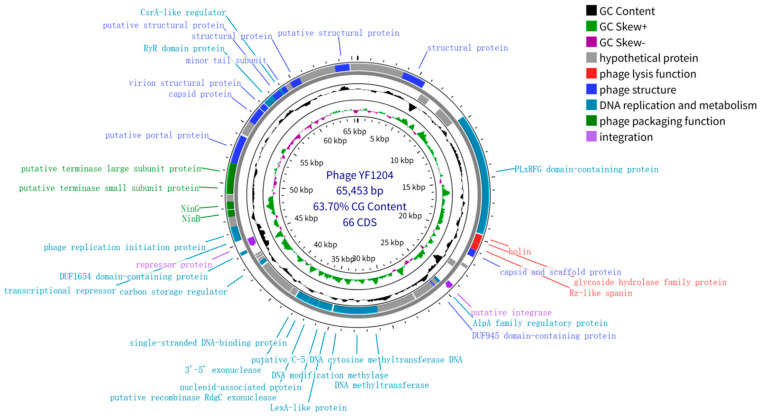
Diagram of the whole-genome pattern of the phage YF1204.

The whole genome of phage YF1204 was sequenced and determined to be a double-stranded DNA molecule of 65,453 bp with a GC content of 63.70% (Figure 2). The genome was predicted to contain 66 open reading frames (ORFs), and no tRNA genes were identified. Functional annotation categorized these ORFs into several modules typical of temperate *siphoviruses*: structural proteins, DNA packaging, DNA replication and metabolism, lysis, lysogeny/regulation, and hypothetical proteins. No homologs of virulence genes, resistance genes, or toxin-encoding sequences were identified in the YF1204 genome.

The DNA packaging module is located in a contiguous cluster and includes genes encoding the putative terminase small (TerS) and large (TerL) subunits, followed by the putative portal protein. The TerS and TerL proteins are essential for recognition of viral DNA and ATP-driven translocation into the procapsid, a process conserved among *Caudovirales* [23]. Downstream of the portal gene, a module encoding structural proteins was identified, including the major capsid protein, scaffolding protein, virion structural protein, and minor tail subunit. This modular organization of structural genes is characteristic of *siphoviral* genomes and facilitates coordinated assembly of the virion [24,25]. It is worth mentioning that the lysis module of YF1204 contains a holin and a glycoside hydrolase family protein, suggesting a two-component system for host cell lysis. Holins form pores in the cell membrane that allow endotoxin to enter the peptidoglycan layer [26]. Near this module, a gene encoding RZ-like spanin was identified. The spanin complex is involved in destroying the outer membrane of gram-negative hosts and completing the lysis process [17,27,28]. The presence of an intact spanin-holin-endolysin system is consistent with the ability of YF1204 to efficiently lyse *P. aeruginosa*.

A cluster of genes associated with DNA metabolism was identified. Notably, genes encoding a putative C-5 DNA cytosine methyltransferase and a DNA modification methylase were present, which may be involved in host DNA modification or phage DNA protection [29,30]. Furthermore, a LexA-like protein homologue was found. In bacteria, LexA is a key repressor of the SOS response, and its presence in phage genomes may indicate a role in modulating the host’s DNA damage response to benefit phage replication [31].

Consistent with its temperate phenotype, the YF1204 genome encodes a putative integrase of the tyrosine recombinase family, which mediates site-specific integration into the host genome [32,33]. Adjacent to the integrase, an AlpA family regulatory protein was identified. AlpA is a master transcriptional regulator in some temperate phages that controls the switch between lysogenic and lytic cycles [34,35]. These genetic features strongly support the capability of YF1204 to establish lysogeny. A significant portion (approximately 42%) of the YF1204 genome consists of hypothetical proteins with no known homologs in public databases. Among the annotated ORFs, two merit special attention due to their novelty: a PLxRFG domain-containing protein and a DUF1654 domain-containing protein. The PLxRFG domain is associated with some phage regulatory proteins, though its precise function remains uncharacterized. The DUF1654 domain is also of unknown function but has been identified in other phage genomes, potentially involved in protein–protein interactions or nucleic acid binding [36,37]. Their presence underscores the genetic novelty of YF1204.

**Figure 3:**
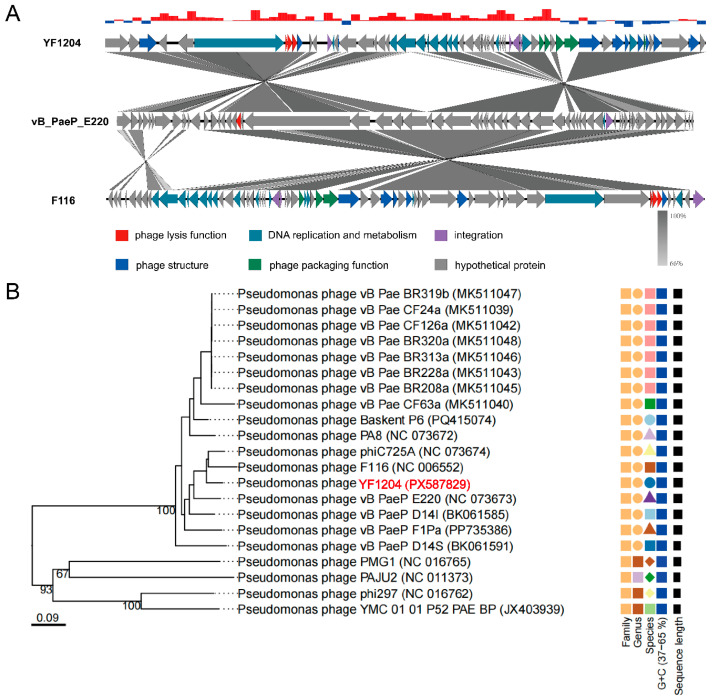
(**A**) Comparative genome analysis and (**B**) phylogenetic tree analysis of phage YF1204.

To elucidate the genomic relatedness and evolutionary position of YF1204 among known *Pseudomonas* phages, a comparative genomic analysis and a whole-genome-based phylogenetic analysis were performed. Genomic analysis using BLASTn revealed that YF1204 shared a modular genome organization with other *Pseudomonas*-infecting *siphoviruses*, particularly with *Pseudomonas* phage F116 (GenBank: NC 006552) and vB_PaeP_E220 (GenBank: NC 073673) (Figure 3A). The genomes of these three phages exhibit conserved synteny in functional modules responsible for lysogeny, DNA replication, packaging, and structural assembly. Notably, the integration module containing the putative integrase gene is located in a similar genomic region across all three phages, supporting a temperate lifestyle. However, YF1204 possesses a distinct arrangement of several hypothetical protein genes and regulatory elements, suggesting potential functional diversification.

Phylogenetic reconstruction was conducted using Viptree based on whole-genome sequence similarity, which provides a robust overview of phage relatedness across entire genomes. The resulting tree (Figure 3B) placed YF1204 within a distinct cluster that includes *Pseudomonas* phages vB_PaeP_E220 and F116, corroborating the findings from the synteny analysis. This clade further grouped with other members of the *F116-like* virus genus, such as vB_PaeP_D141, PA8, and phiC725A [38]. The branching pattern indicates that YF1204 shares a most recent common ancestor with vB_PaeP_E220 and F116, yet occupies a separate branch, supporting its classification as a novel member within this genus.

Together, these findings collectively indicate that YF1204 is a temperate *siphophage* phylogenetically affiliated with the *F116-like* phage group, characterized by a conserved core genome architecture while possessing unique genetic elements that may confer specific functional traits [38,39,40]. Furthermore, these analyses expand our understanding of phage diversity and potential for therapeutic exploitation.

### 3.2. Growth Kinetics, Stability, and In Vitro Antibacterial Activity of Phage YF1204

**Figure 4:**
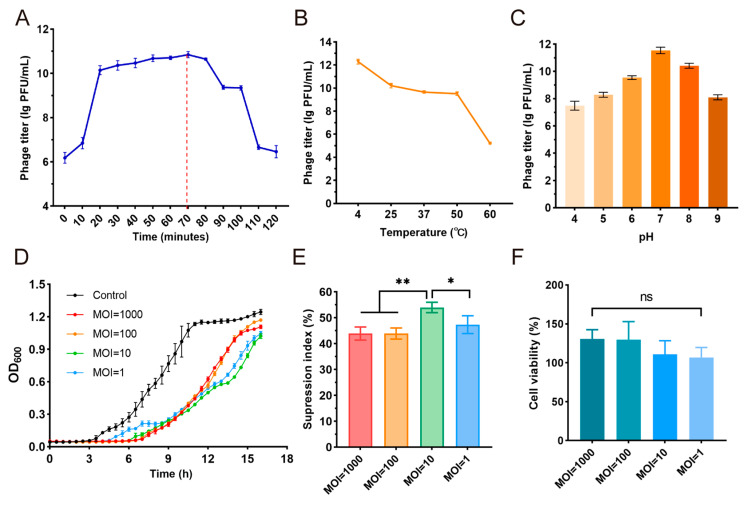
One-step growth curve and biological characteristics of phage YF1204. (**A**) One-step growth curve, (**B**) temperature stability, (**C**) pH stability, (**D**) antibacterial kinetics curve, (**E**) suppression index and (**F**) cytotoxicity test. (* *p* < 0.05, ** *p* < 0.01, ns represents no significance).

The optimal MOI, one-step growth and stability of phage YF1204 were determined by the double-layer agarose plate method. The YF1204 phage titer peaked at an MOI of 10 and was selected for further experiments (Figure S1). The one-step growth curve of bacteriophage YF1204 revealed a typical lytic cycle with distinct latent, rise, and plateau phases (Figure 4A). The latent period lasted approximately 20 min, after which the phage titer increased rapidly, indicating the onset of host cell lysis and virion release. The rise phase continued until approximately 70 min post-infection, after which the phage titer stabilized. Furthermore, the burst size of phage YF1204 was about 50 average progeny per infected cell.

The environmental stability of YF1204 was assessed under varying temperature and pH conditions. The phage exhibited high thermal stability, retaining full infectivity after incubation for 1 h at temperatures ranging from 4 °C to 50 °C (Figure 4B). A significant reduction in titer was observed at 60 °C. These results indicate that YF1204 is relatively thermostable under moderate temperatures, which may support its persistence in varied environmental conditions. Furthermore, the phage exhibited maximal stability in the neutral to slight range (pH 6.0–8.0), with titers remaining above 10^8^ PFU/mL (Figure 4C). These results indicate that YF1204 is resilient under moderate environmental conditions, which may support its persistence in natural settings and potential utility in applications within physiological or near-neutral pH environments.

The antibacterial efficacy of phage YF1204 against its host bacterium was evaluated across a range of MOI values (MOI = 1, 10, 100, and 1000) over a 16 h period. The time-kill curves revealed a clear MOI-dependent inhibitory effect (Figure 4D). At higher MOI values (MOI = 100 and 1000), rapid suppression of bacterial growth was observed within the first 2 h, indicating a strong early bacteriolytic activity. In contrast, at lower MOI values (MOI = 1 and 10), bacterial growth was initially similar to the control but was progressively inhibited after 4 h, suggesting a delay in phage amplification and lysis.

To quantitatively compare the overall inhibitory effect, the suppression index was calculated based on the area under the bacterial growth curve (AUC) over 16 h (Figure 4E). The highest suppression index was observed at MOI = 10, which achieved approximately 53% inhibition of bacterial growth. Although MOI = 100 and 1000 exhibited stronger early activity, their overall suppression indices were slightly lower than that of MOI = 10, possibly due to early resource depletion or the emergence of partial resistance at extremely high phage concentrations. MOI = 1 showed the weakest inhibition, consistent with its delayed lytic activity. These results indicated that MOI = 10 represented the optimal infection dosage for YF1204 under the tested conditions, balancing rapid inhibition with sustained antibacterial efficacy.

Furthermore, to evaluate the biosafety of phage YF1204 for potential therapeutic applications, its cytotoxic effect on human monocytic THP-1 cell was assessed using a CCK-8 assay after 24 h of co-culture at various phage concentration (10, 100, and 1000 PFU/cell). As shown in Figure 4F, no significant reduction in cell viability was observed at any of the tested MOI values compared to the mock-treated control group. Cell viability remained consistently above 95% across all phage-treated groups, including at the highest concentration of 1000 PFU/cell. These results indicated that YF1204 exhibited negligible cytotoxicity toward mammalian cells under the experimental conditions, initially confirming the cellular safety of YF204 for therapeutic applications [41].

### 3.3. Host Range Analysis

**Figure 5:**
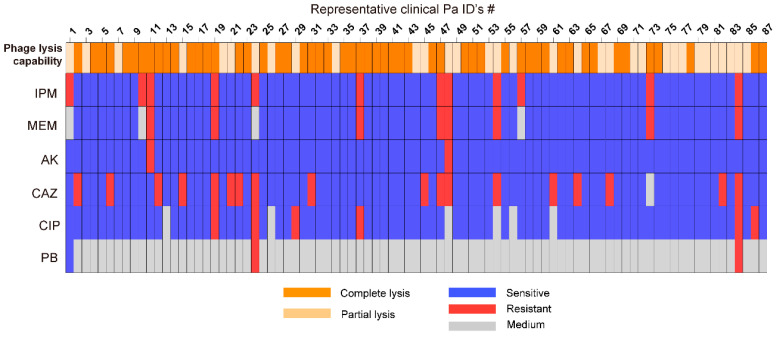
Profile of host range and antibiotic susceptibility analysis. (Imipenem for IPM, meropenem for MEM, amikacin for AK, ceftazidime for CAZ, ciprofloxacin for CIP, and polymyxin B for PB. Blue represents sensitive, red represents resistant, and gray represents medium).

The host range of phage YF1204 was evaluated against a collection of 210 clinical *P. aeruginosa* isolates. Among these, 87 isolates (41.4%) produced visible plaques on double-layer agarose plates and were further categorized based on lysis efficiency (Figure 5). Of the plaque-positive strains, complete lysis (indicated by orange shading) was observed in 62.0% (60/87) of isolates, while 37.9% (33/87) exhibited partial lysis (indicated by light orange shading). A total of 34 CRPA were present, among which 12 strains could produce plaque, with a lysis ratio of 35.29% (12/34), and 20.58% were completely lysed (7/34).

Antibiotic susceptibility testing of the 87 phage-sensitive isolates revealed a high prevalence of multidrug resistance. Notably, all carbapenem-resistant isolates (imipenem/IPM and meropenem/MEM resistant) remained fully susceptible to amikacin and were efficiently lysed by YF1204 (complete or partial lysis). This consistent phenotype-carbapenem resistance, amikacin sensitivity, and phage susceptibility highlighted a promising target population for combination therapy.

### 3.4. Phage–Antibiotic Combination Inhibits CRPA

**Figure 6:**
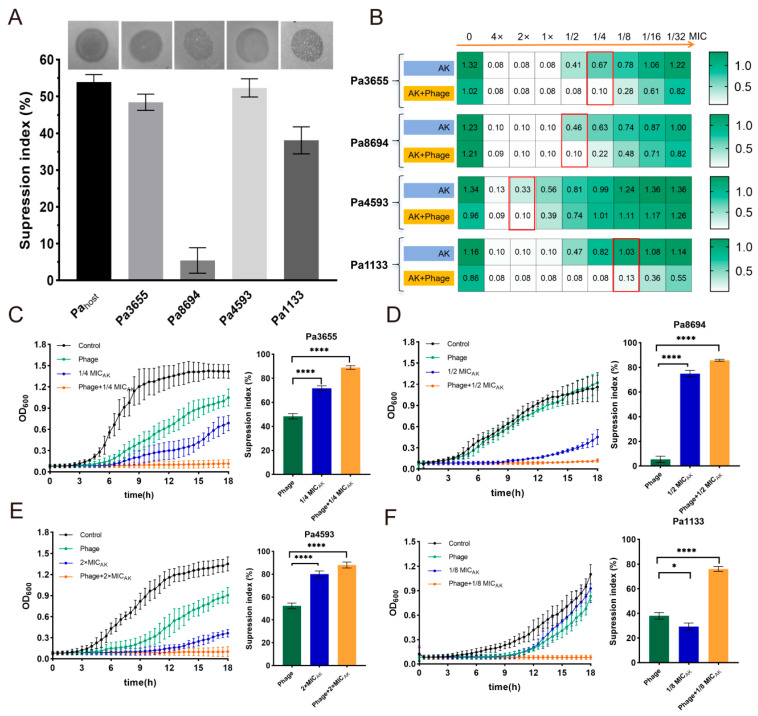
In vitro phage YF1204-AK synergy against clinical CRPA isolates. (**A**) Phage antibacterial index; (**B**) screening of synergy effect by checkerboard method. Dynamic inhibition curve and suppression index of phage YF1204-AK combination against (**C**) strain Pa3655 (**D**) Pa8694 (**E**) Pa4593 and (**F**) Pa1133. (* *p* < 0.05, **** *p* < 0.0001).

Based on the above correlation, to quantitatively assess the interaction between phage YF1204 and conventional antibiotics, checkerboard assays were performed against four clinical CRPA isolates, with bacterial growth measured by OD_600_ after 18 h of coincubation. First, the suppression index of the phage against the host strain and different CRPA strains was determined, and the results showed that the suppression index was 53% for the host strain, 48% for Pa3655, 6% for Pa8694, 52% for Pa4593, and 37% for Pa1133, respectively (Figure 6A). This indicated that the phage YF1204 had a comparable inhibition ability to the host strain except for strain Pa8694.

Furthermore, we systematically screened for synergistic partners against clinical CRPA isolates among frontline antibiotics, including meropenem (MEM), amikacin (AK), ceftazidime (CAZ), ciprofloxacin (CIP), or polymyxin B (PB). Direct comparison of combination efficacy revealed a striking difference between antibiotic classes. Combinations of YF1204 with MEM, CAZ, CIP, or PB did not exhibit enhanced suppression (Figure S2). For these antibiotics, the OD_600_ values in combination wells were typically comparable to, or in some cases higher than, the values observed with the more effective single agent.

Crucially, we identified AK, an aminoglycoside antibiotic for treating *Pseudomonas* infection, as an effective synergistic partner of phage YF1204 (Figure 6B). For each strain, the combination of a sub-inhibitory concentration of YF1204 with serially diluted AK resulted in significantly lower OD_600_ readings compared to AK alone across multiple concentration points, indicating markedly enhanced bacteriostatic activity. For Pa3655, at AK concentrations corresponding to 1/4 MIC, where single-agent AK permitted substantial growth (OD_600_ = 0.67), the addition of phage suppressed growth to near-background levels (OD_600_ = 0.10). For Pa8694, a similar pattern was observed. At the 1/2 MIC AK concentrations, growth was reduced from OD_600_ = 0.46 (AK alone) to OD_600_ = 0.10 in combination. While strain Pa4593 showed higher baseline growth in the presence of AK alone at lower concentrations, the phage–AK combination still demonstrated a clear inhibitory advantage, particularly at intermediate concentrations. The most pronounced synergy was observed against Pa1133. Concentrations of 1/8 MIC AK that alone supported robust growth (OD_600_ = 1.03) were rendered completely or strongly inhibitory in the presence of phage (OD_600_ reduced to 0.13).

To dynamically assess the enhanced efficacy of the phage–antibiotic synergy, time-killing assays were conducted and the overall antibacterial effect was quantified using the suppression index, derived from the AUC over 18 h. Against all four CRPA strains, the combination of phage YF1204 with sub-inhibitory concentrations of AK (1/8×, 1/4×, or 1/2× MIC) resulted in a dramatically superior outcome compared to any monotherapy. The combination therapy achieved suppression indexes ranging from 79% to 89% across the four CRPA strains, significantly exceeding those of phage alone (6–53%) or different concentrations of AK alone (29–76%) (Figure 6C–F). The time-killing curves revealed that while phage alone often caused an initial decline in bacterial density followed by regrowth, and AK alone at these low concentrations had minimal effect, their combination led to rapid and sustained suppression of bacterial growth.

This visual superiority was conclusively quantified by the suppression index. For each strain and at every sub-MIC AK concentration tested, the combination therapy achieved a significantly higher suppression index (*p* < 0.0001) than either phage or AK alone (Figure 6C–F). For example, against strain Pa1133, the combination with 1/8× MIC AK yielded a significant suppression (~80%), vastly exceeding the modest effect of either agent alone (Figure 6F). Even when combined with a supra-inhibitory antibiotic concentration (2× MIC) against strain Pa4593, the addition of phage further significantly increased the suppression index compared to the antibiotic alone, indicating additive or synergistic effects across the antibiotic concentration spectrum (Figure 6E). These kinetic data, quantified via suppression indices, demonstrated that the synergy between phage YF1204 and AK transformed a sub-therapeutic antibiotic dose into a potent, long-lasting bactericidal regimen, effectively preventing the bacterial recovery commonly observed with phage monotherapy.

**Figure 7:**
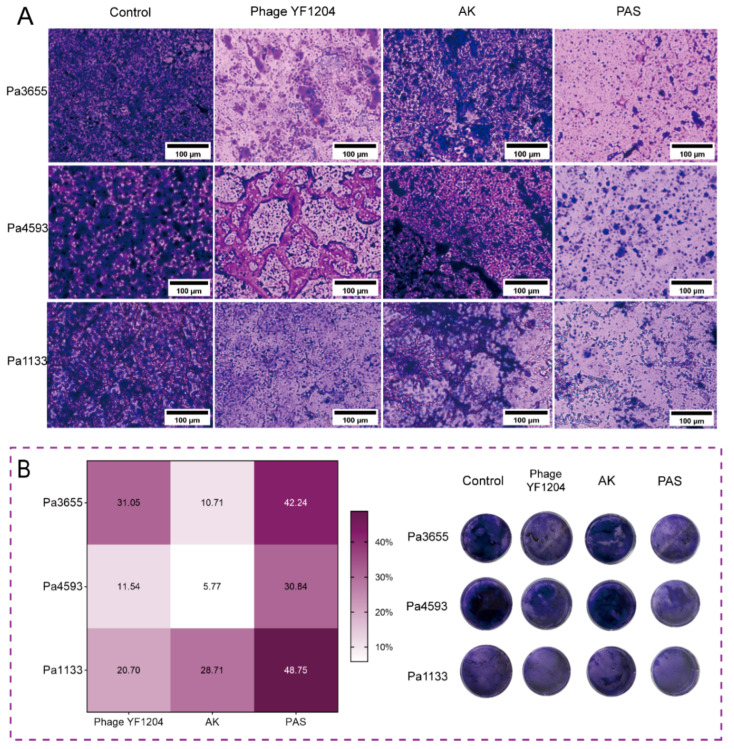
Synergistic inhibition of biofilm formation in CRPA by phage YF1204 and AK. (**A**) Microscopic images of the biofilm formation inhibition assay. (**B**) Statistical analysis heat map based on the the biofilm formation inhibition ratio (%), and digital photos of the biofilm.

Additionally, the ability of phage YF1204 and AK, and their combination to prevent biofilm formation was assessed against three CRPA strains using crystal violet staining and microscopy. Microscopic images revealed distinct structural differences among treatment groups (Figure 7A). The untreated control groups displayed dense, confluent biofilm architectures. Monotherapy with either phage YF1204 or AK resulted in visibly reduced biofilm biomass and increased structural disruption. However, the most pronounced effect was observed in the phage–AK synergy (PAS) groups, which showed sparse, patchy biofilm formation with significantly diminished bacterial aggregation on the abiotic surface. Biofilm formation inhibition was quantified by calculating the percentage reduction in crystal violet staining relative to the untreated control (Figure 7B). The PAS consistently yielded the highest biofilm formation inhibition ratios for all three strains (42.24% for Pa3655, 30.84% for Pa4593, 48.75% for Pa1133). These values were substantially higher than those achieved by either agent alone. For instance, against the highly susceptible strain Pa1133, the combination’s inhibition ratio (~49%) exceeded the sum of the individual effects of phage (~21%) and AK (~29%), suggesting a synergistic interaction preventing biofilm establishment. The digital images showed visibly fainter crystal violet staining in the combination treatment wells compared to all other groups, confirming the superior biofilm-suppressive activity of the PAS regimen at a macroscopic scale. These results demonstrated that the synergistic activity of phage YF1204 and AK extended beyond planktonic cells to effectively inhibit the formation of structured biofilms, a key virulence trait associated with persistent and recalcitrant CRPA infections.

## 4. Discussion

The rising prevalence of multidrug-resistant *P. aeruginosa*, particularly carbapenem-resistant strains, poses a grave challenge to global public health, necessitating the exploration of alternative therapeutic strategies [42]. This study isolated and characterized a novel temperate phage, YF1204, and systematically evaluated its potential, both alone and in combination with conventional antibiotics, against clinical CRPA isolates. Our findings demonstrated that YF1204 represented a new member of the *F116-like* genus within the *Siphoviridae* family, exhibited a moderate and potent lytic spectrum against CRPA, and synergized powerfully with AK to suppress both planktonic and biofilm-associated bacterial populations.

The morphological and genomic analyses conclusively place phage YF1204 within the well-studied *F116-like* virus genus. Its icosahedral head and long non-contractile tail are hallmarks of siphoviruses. The genome architecture, featuring conserved modules for lysogeny (integrase, *LexA-like* repressor), DNA replication, and structural proteins, aligns with the genetic blueprint of temperate *F116-like* phages [40,43]. Importantly, whole-genome screening confirmed that YF1204 does not carry any known virulence or antimicrobial resistance genes, mitigating a primary safety concern associated with temperate phage therapy. This temperate lifestyle is corroborated by the presence of an integration module and the observed sustained suppression in time-killing assays, where a subpopulation may enter a persistent state. The high burst size (50 PFU/cell) of YF1204 may be attributed to highly efficient replication and lysis mechanisms, a trait that could translate to potent in vivo activity if the phage is used in a lysis-competent, engineered, or conditionally induced form for therapy [13,44].

A critical finding was the complementary susceptibility pattern between phage YF1204 and AK. While the phage lysed a significant proportion (41.4%) of clinical isolates, it showed particular efficacy against strains that were resistant to carbapenems yet remained AK-susceptible. This correlation formed the rationale for testing a combination strategy. Our checkerboard and time-killing assays unequivocally identified AK, among several tested antibiotics, as the sole synergistic partner for YF1204. The combination achieved what neither agent could accomplish alone: rapid initial killing followed by sustained suppression without regrowth. This can be explained by a two-pronged mechanistic synergy. Firstly, AK, an aminoglycoside, may enhance phage infection by perturbing the bacterial outer membrane, increasing phage receptor accessibility or facilitating DNA injection [45]. Secondly, the phage’s lytic action could eliminate bacterial subpopulations that are inherently tolerant to sub-MIC AK, while simultaneously drastically lowering the population density, thereby reducing the probability of selecting for spontaneous AK-resistant mutants [46]. The therapeutic promise of this combination extended beyond planktonic cells to the biofilm state, a major determinant of CRPA persistence. We found that YF1204 and AK acted synergistically to inhibit biofilm formation, with the combination achieving significantly greater biomass reduction than the sum of individual effects. This anti-biofilm activity likely stems from the phage disrupting the nascent biofilm structure by lysing anchor cells, while AK inhibits protein synthesis in adjacent, matrix-exposed bacteria [47]. Preventing biofilm establishment is clinically significant, as it could mitigate device-related colonization and chronic infections.

Single-agent antibiotic treatment is highly susceptible to driving further evolution of resistance, leading to the failure of the last effective drugs. Therefore, we explored the use of phage YF1204 in combination with AK in order to: (1) take advantage of their synergistic effect to achieve a more rapid and complete bactericidal effect than either drug alone; (2) significantly reduce the risk of bacterial resistance to AK through multi-mechanism attack, thereby extending the clinical lifespan of this key antibiotic; (3) to provide a prospective combination treatment strategy for more drug-resistant strains that may emerge in the future.

Last but not least, while our in vitro findings are compelling, several limitations must be acknowledged. First, the temperate nature of YF1204 necessitates caution, as lysogeny could potentially facilitate horizontal gene transfer. Future work should involve the construction of lytic mutants using genetic engineering (e.g., through deletion of the integrase gene and virulence gene) for therapeutic development. Second, we acknowledged that the current study represented a critical in vitro proof-of-concept. Any future in vivo applications would require additional regulatory approvals and safety studies in appropriate animal models, as outlined by current phage therapy guidelines. During therapy, bacterial isolates from treated patients would be monitored for lysogeny or horizontal gene transfer events. Third, the potential for the emergence of phage resistance, although mitigated by the combination, should be monitored in long-term passage experiments. Finally, exploring the combination’s effect on host immune responses and its efficacy against intracellular bacteria would provide a more comprehensive preclinical picture.

## 5. Conclusions

This study isolated and characterized a novel temperate phage, YF1204, belonging to the *F116-like* group, which exhibited a moderate host range and efficient lytic activity against clinical CRPA isolates. The combination of YF1204 with amikacin (AK) demonstrated significant synergistic effects in vitro, potently inhibiting planktonic bacterial growth and biofilm formation, preventing regrowth at sub-inhibitory antibiotic concentrations. This specificity likely stems from AK’s mechanism as a protein synthesis inhibitor, which may compromise the maintenance of lysogeny by interfering with phage repressor production. These findings highlight the potential of this phage–antibiotic combination as a promising therapeutic strategy against recalcitrant, biofilm-associated infections caused by multidrug-resistant *P. aeruginosa*. Further studies are warranted to validate its efficacy in vivo.

## Data Availability

The original contributions presented in this study are included in the article and Supplementary Material. Further inquiries can be directed to the corresponding authors.

## Supplementary Materials

The following supporting information can be downloaded at: https://www.mdpi.com/article/10.3390/microorganisms14030549/s1, Figure S1: The MOI of phage YF1204; Figure S2: Screening of synergy effect of meropenem (MEM), ceftazidime (CAZ), ciprofloxacin (CIP), or polymyxin B (PB) against clinical CRPA isolates (Pa3655, Pa8694, Pa4593, Pa1133) by checkerboard method.
