# Supplementary material for: Synergistic Efficiency of a Novel Temperate Phage YF1204 and Amikacin Against Carbapenem-Resistant Pseudomonas aeruginosa and Its Biofilms
Source: Microorganisms. 2026 Feb 27;14(3):549. doi: 10.3390/microorganisms14030549 (PMC13028781; doi:10.3390/microorganisms14030549)
Supplement: Supplementary file 1 [file microorganisms-14-00549-s001.zip › microorganisms-4150640-supplementary.pdf]

Supplementary Data

Synergistic Efficiency of a Novel Temperate Phage YF1204 and Amikacin Against Carbapenem-Resistant *Pseudomonas aeruginosa* and Its Biofilms

Yinfeng Yang [a], Noura M. Bin Yahia [b], Yafei Pan [a], Zhaoxia Ran [a], Jing Yang [a], Yanhui Yang [b,*] , Gang Li [a,**]

[a] *Center of Medical Laboratory, General Hospital of Ningxia Medical University; Ningxia Key Laboratory of Clinical and Pathogenic Microbiology, General Hospital of Ningxia Medical University, Yinchuan 750004, China*

[b] *Ningxia Key Laboratory of Infectious and Immunity, School of Basic Medical Sciences, Ningxia Medical University, Ningxia Key Laboratory of Clinical and Pathogenic Microbiology, General Hospital of Ningxia Medical University, Yinchuan 750004, China*

[*] Corresponding author. *E-mail addresses:* yyhysf@163.com (Y. Yang)

[**] Corresponding author. *E-mail addresses:* ligang@nyzy.com.cn (G. Li).

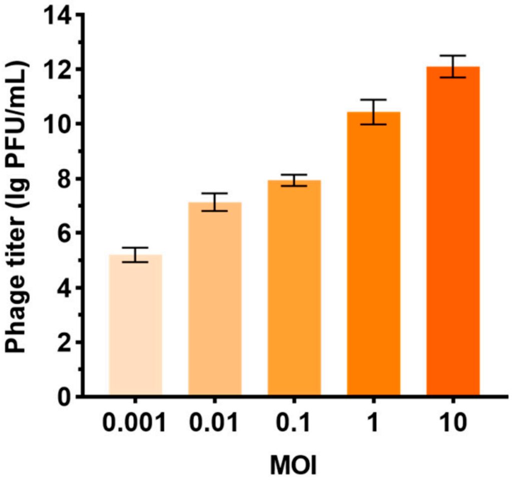

Figure S1. The MOI of phage YF1204.

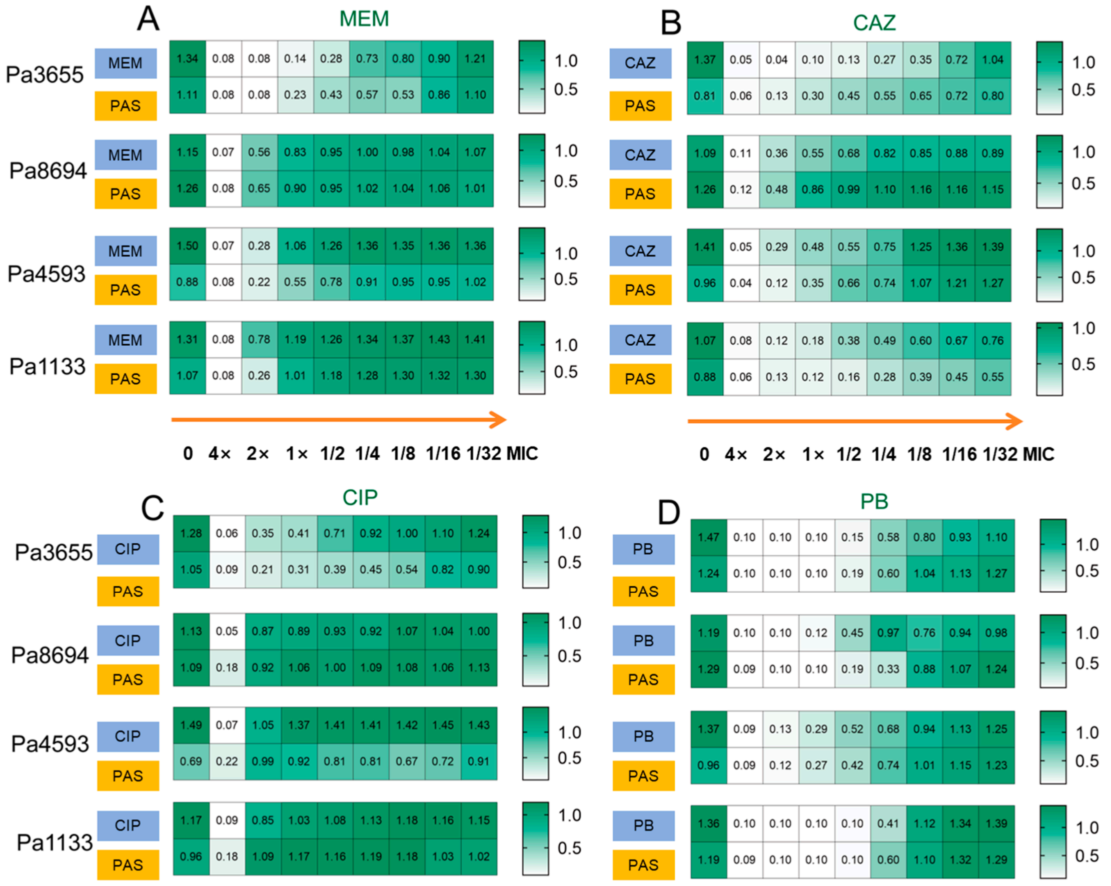

Figure S2. Screening of synergy effect of meropenem (MEM), ceftazidime (CAZ), ciprofloxacin (CIP), or polymyxin B (PB) against clinical CRPA isolates (Pa3655, Pa8694, Pa4593, Pa1133) by checkerboard method. The horizontal axis represents

different antibiotic concentrations, PAS rows represent phage combined with different concentrations of antibiotics, and the OD_{600} values were marked in each well (n=3).
